# College EFL teachers’ demotivation to conduct research: A dynamic and ecological view

**DOI:** 10.3389/fpsyg.2022.1071502

**Published:** 2023-01-19

**Authors:** Xiaobin Ren, Fen Zhou

**Affiliations:** School of Foreign Languages and Affairs, Hubei Business College, Wuhan, China

**Keywords:** EFL teachers, EFL teacher education, motivation, research competence, demotivation to conduct research

## Abstract

Competences for conducting research is vitally important for college EFL teachers’ career development, but many college English teachers are demotivated in academic research. To investigate teachers’ motivation on academic activities, this study firstly explored motivational changes of college EFL teachers (mean age 37.39, SD 9.77) for conducting research in their teaching career, and then delved into the factors underlying their demotivation by sending questionnaires. In the end, several English teachers and officers managing research projects were interviewed to elicit solutions to overcome EFL teachers’ demotivation to conduct research. This study found that college EFL teachers had large possibilities to suffer from demotivation to conduct research. Exploratory factor analysis indicated five factors causing their demotivation, including weak research ability, negative emotions and attitudes, poor research surroundings, research management problems and insufficient resources. Thematic analysis demonstrated that ecological solutions should be taken by different stakeholders in EFL teachers’ working ecology, including universities, research communities, government, and publishers. This study problematized the static view on teachers’ demotivation to conduct research and provided some insights and implications for reasons and solutions for demotivation.

## 1. Introduction

In the fierce competition of higher education in the world, academic research intensity is one of the essential indicators of the overall educational quality of universities (Mägi and Beerkens, 2016). To improve the competitive edge in the university rankings, many universities encourage their teaching staff to conduct academic research. For example, numerous universities in China tend to link teachers’ promotions and incentives with their research output and hence urge college teachers to pursue scholarship (Zhao, 2015; Peng and Gao, 2019). From the perspective of English as a foreign language (EFL) teachers, conducting research could deepen their understanding of EFL learners, teachers, teaching practice and other crucial elements in their career (Gao and Chow, 2011; Borg and Liu, 2013). Therefore, college EFL teachers, an important group in college teachers, should conduct academic research for the sake of themselves, their students and the universities they are working in. However, it is not uncommon to find that many college EFL teachers spend fairly limited time on academic research (e.g., see Chen and Wang, 2013) even though encouraged by universities with various benefits. Consequently, many college EFL teachers’ research capacities and publications were rather limited (Bai and Hudson, 2011; Wang and Han, 2011). Considering the importance of research to EFL teachers, the scant investments, competences, and publications might hinder their career development and their students’ English learning.

Currently, English teachers’ demotivation in their teaching career has been noticed among several researchers in different countries (e.g., Yaghoubinejad et al., 2017; Nagamine, 2018; Khanal et al., 2021; Kim and Kim, 2022), but a paucity of studies distinguished their (de)motivation of English teaching from that of conducting academic research or even shift their focus to EFL teachers’ research intensity (Yuan et al., 2016). Indeed, several studies investigated English teachers’ motivation to conduct research (e.g., Borg and Liu, 2013), but the dark side of motivation (i.e., demotivation) was neglected, and hence the factors underlying EFL teachers’ demotivation to conducted research (hereinafter, DTCR) and corresponding solutions were rarely explored. Besides, although there exist little research focusing on EFL teachers’ research behaviors, few researchers investigated the dynamic and flexible process of (de) motivation in their long teaching career path.

Based on these considerations, this study explored the dynamic processes of college EFL teachers’ (de)motivation to conduct research, investigated the factors underlying their DTCR, and delved into the solutions to overcome it. This study could help to understand EFL teachers’ DTCR better and provide suggestions for stakeholders to overcome DTCR.

## 2. Literature review

### 2.1. Motivation, amotivation, and demotivation

Studies on motivation increased gradually after Gardner and Lambert (1972) proposed their classic concepts of integrative motivation and instrumental motivation. However, there is no universally accepted definition on motivation until now because of its complexity (Han and Yin, 2016). For example, Kleinginna and Kleinginna (1981) classified existing motivation definitions into nine categories in light of their focuses. By drawing upon various definitions, Dörnyei and Ushioda (2011) concluded that direction and magnitude of human behavior were the two dimensions most researchers agreed for defining motivation. In other words, motivation stipulated the reasons why individuals are determined to do an activity, the length they will sustain the activity and the efforts they are going to make for this activity.

Apart from motivation, its dark sides were also frequently investigated in related studies. Currently, amotivation and demotivation were often applied to express the meaning that people work or study with decreased motivation in existing studies (e.g., Balkis, 2018; Wu et al., 2020; Albalawi and Al-Hoorie, 2021). Amotivation was firstly used in self-determination theory (Deci and Ryan, 1985), where it was defined as the relative absence of motivation that is caused by an individual’s feelings of incompetence and helplessness when faced with an activity. For example, when encountered with unmasterable situations, people would suffer from helplessness, depression, listlessness, and self-disparagement, which would lead to the decrease of motivation. Compared with amotivation, demotivation was more frequently used by researchers in second language acquisition (SLA) field (Ren, 2022). Dörnyei and Ushioda (2011, p. 139) once defined demotivation as “specific external forces that reduce or diminish the motivational basis of a behavioral intention or an ongoing action.” They compared the two terms of amotivation and demotivation, and thought amotivation is related to some unrealistic expectations, while demotivation is linked with specific external reasons. In fact, neither the definitions of the two terms could cover the complex phenomenon of motivation decrease among college EFL teachers. For example, several researchers (e.g., Sakai and Kikuchi, 2009; Clare et al., 2019) disagreed with Dörnyei and Ushioda (2011)’s definition of demotivation and included both internal and external factors when they were investigating demotivation. In this demotivational study, both the scopes of “demotivation” and “amotivation” were included. However, considering the terminology of “demotivation” was more frequently used in L2-related research articles than “amotivation” (see Table 1), this paper applied the term “demotivation,” though the definition was slightly different from that in the great book of Dörnyei and Ushioda (2011).

**Table 1:** Frequency of results from google scholar.^a^

Words keyed in	Frequency
Demotivation	About 82,600
Amotivation	About 33,700
Demotivation in language teaching	About 31,200
Amotivation in language teaching	About 11,700

a Data was retrieved on 3/September/2022.

Based on the above considerations, college EFL teachers’ DTCR was defined as low or decreased investment and engagement in conducting research because of both internal and external factors in this study. This definition was drawn upon and synthesized from the studies conducted by Clare et al. (2019), Sakai and Kikuchi (2009), Pishghadam et al. (2021), Deci and Ryan (1985), and Dörnyei and Ushioda (2011), etc.

### 2.2. Teacher demotivation

There were several studies conducted to explore EFL teachers’ demotivation in various educational stages. For example, Yaghoubinejad et al. (2017) found the major demotivating factors for Iranian junior high school English teachers included lack of social recognition and respect, few adequate rewards, lack of supports or understanding regarding English education, and a large number of students in a single English class. Kim et al. (2014) compared the differences of demotivating factors between English teachers (in elementary and junior high schools) in China and South Korea. Their study indicated that large English class size was the common detrimental factor for English teachers in the two countries. But there existed some different demotivating factors for English teachers in the two countries. For example, only English teachers in China perceived “excessive interference or expectations of school parents” was a demotivating factor, while “large amounts of administrative tasks and students’ lack of interest” were found to be the demotivating factors for English teachers in South Korea. Similarly, Baniasad-Azad and Ketabi (2013) compared the factors causing English teachers’ demotivation in Iran and Japan, and found some analogous and different underlying factors. In addition, Ozturk (2021) went even further and investigated only one ability of English teachers (i.e., assessment) in the teaching process and uncovered the reasons for EFL teachers’ demotivation to be more assessment literate in Turkey.

However, most of the existing research only focused on investigating the underlying factors or reasons causing EFL teachers’ demotivation in their language teaching, but few explored their DTCR. Therefore, research on EFL teachers’ DTCR should be strengthened. First, teachers’ teaching and research are distinct in several dimensions, though they are often interrelated with each other (Cochran-Smith and Lytle, 1990; Liu et al., 2010). Research on teachers’ demotivation to teach might not provide enough information for understanding their DTCR. Hence, the different properties between teaching and research require studies specifically on research demotivation. In addition, teaching and research are the two most fundamental functions of universities, and they are the two sides of college teachers’ identities (Liu et al., 2010). Therefore, both demotivation of teaching and research deserve attentions in studies. Third and practically, universities tend to encourage and press their teaching staff to conduct academic research to gain competitiveness in the world university rankings and other various evaluations (Mägi and Beerkens, 2016). These practical research policies, regulations or requirements need more attentions paid on EFL teachers’ DTCR. For example, the factors causing EFL teachers’ DTCR and the solutions to overcome this problem and remotivate them should be investigated.

People’s (de)motivation was a changeable and dynamic process (e.g., see Waninge et al., 2014; Song and Kim, 2016; Zheng et al., 2020; Wang and Littlewood, 2021), but current studies seldom considered the fluctuation of teachers’ motivation of conducting research. Most of the motivation studies were conducted to delve into the factors underlying their motivational behaviors (e.g., see Bai and Hudson, 2011; Borg and Liu, 2013; Yuan et al., 2016), but little research paid attention to the motivation tendency in EFL teachers’ long teaching career. Therefore, to have a better understanding of the overall characteristics of this dynamic phenomenon, studies should be conducted to investigate the changing process of EFL teachers’ (de)motivation.

### 2.3. Ecological systems theory

Ecological systems theory (EST) was proposed by Bronfenbrenner (1979), which has changed the approaches for conducting research on the relations between human beings and their surrounding environments among numerous behavioral scientists and sociologists (Ceci, 2006, p. 173). Ecological systems were composed of a set of homocentric and nested sub-systems, including microsystems, mesosystems, exosystems, and macrosystem based on the extent of closeness between a person and the corresponding systems. Microsystems refer to those activities, roles, and interpersonal relations sensed by the progressing human being in a particular setting. Mesosystems are founded on the microsystems, and for a mesosystem, it is composed of the interrelations among two or more settings where the progressing human being directly take part. Exosystems are the settings that are not directly related to the progressing human being. However, those indirect settings could still impact or be impacted by the progressing person through the connecting person or even systems. Macrosystem refers to the setting at the culture or sub-culture levels. The above three lower-order systems are embedded in macrosystem. Macrosystem could be the underlying belief or ideologies for the above three systems (for more details, see Bronfenbrenner, 1979).

Although EST was originally proposed to explain the influences of environment on children’s development (Graves and Sheldon, 2018), this theory has been gradually drawn upon as a theoretical framework to guide the studies of teacher education. For example, Hwang (2014) delved into how ecological contexts influence South Korean teacher educators’ professional development and found global, political, social and institutional contexts could exert impacts on their development. Price and McCallum (2015) applied this theoretical framework to investigate teachers’ well-being and “fitness” among 120 pre-service teachers and concluded that ecological influences were regarded to impact on their well-being and competence to be suitable for sustainable performance. Mohammadabadi et al. (2019) inspected the factors influencing cognition among EFL teachers at various levels and found ecological factors at microsystem, mesosystem, exosystem and macrosystem levels could be the causes underlying their cognition. Therefore, EST could be applied as an appropriate theoretical framework in the research of EFL teachers. In addition, people’s behaviors are usually influenced by different environment factors (e.g., see Rubenstein et al., 2018), indicating that ecological perspectives should be considered when the reasons and solutions for EFL teachers’ DTCR were investigated.

Based on the review of the current studies and theories, this study aims to answer the following research questions:

1. How does college EFL teachers’ motivation for conducting research change in their career?
2. What are the reasons causing college EFL teachers’ DTCR?
3. How to help college EFL teachers overcome their DTCR or remotivate them?

## 3. Methods

Both quantitative and qualitative data were applied to answer the 3 research questions in this study. To collect data, this study applied two instruments among college EFL teachers, including questionnaires and interviews. Totally, 339 English teachers returned the questionnaires, and 15 English teachers or officers accepted the interviews. Exploratory factor analysis and thematic analysis were applied as the data analyzing method to analyze quantitative and qualitative data.

### 3.1. Instruments

**Figure. 1:**
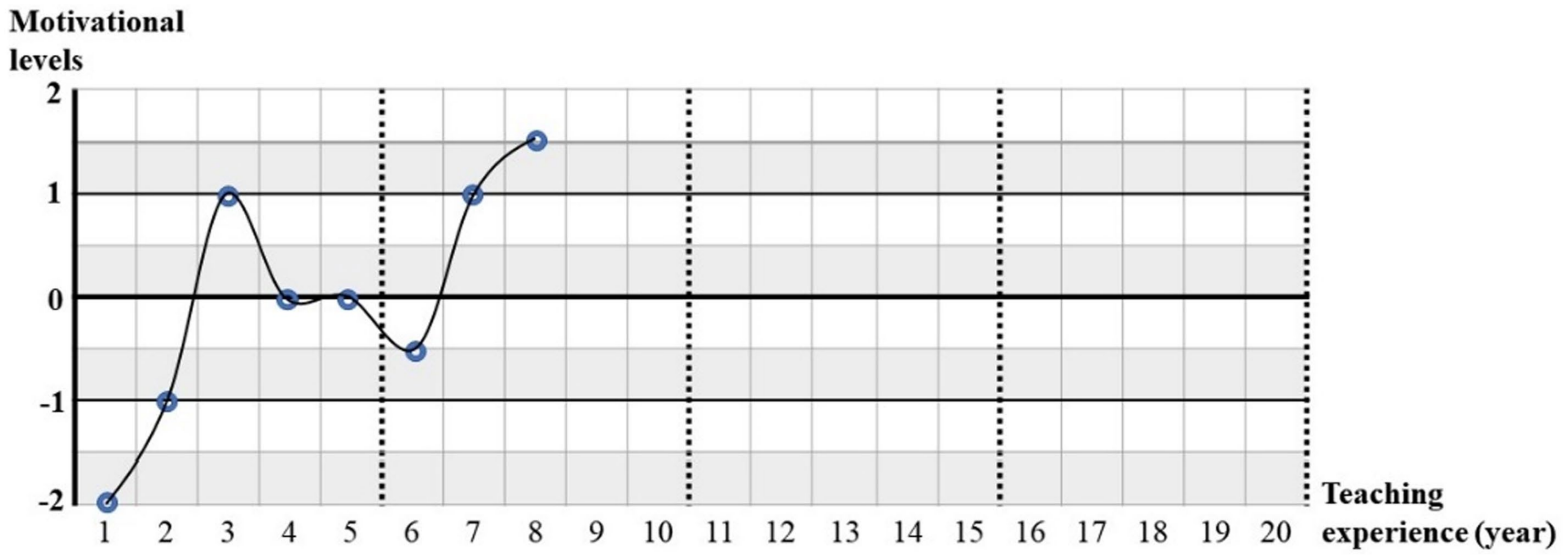
Sample of motivational change graph for academic research. Motivation level 2 = very interesting, 1 = interesting, 0 = neither interesting nor boring, −1 = not interesting, −2 = very boring.

Research instruments applied in this study include questionnaires and semi-structured interviews. The questionnaire included three sections, i.e., basic information (Section 1), identifying question and motivational change graph (Section 2), and 7-point Likert scales (Section 3). The first section aimed to induce the information of college English teachers’ basic information, including age, teaching experience, etc. In the second section, the participants were firstly asked to indicate whether they had experience of DTCR and then to draw lines or carves on the motivation change graph. The motivation change graph was firstly created by Jung (2011) in his investigation of students’ motivational changes and was adopted by Wang and Littlewood (2021) and Song and Kim (2017) when they were delving into the dynamics of students’ demotivation. In this study, this instrument was used to stimulate college EFL teachers to chart their motivation changes over their teaching career. They were guided to dot to indicate their motivational levels from very boring (−2) to very interesting (+2) at a particular year in their teaching experience and then to draw graphs by linking those separated dots. The teachers who had more than 20 years’ teaching experience were only required to display their first 20 years’ experience. In Figure 1, one EFL teacher’s motivational changing track for conducting research was demonstrated. As demonstrated in this curve, this teacher had 8 years’ teaching experience, during which her motivation levels of conducting research fluctuated. Specifically, she was quite demotivated in conducting research at the beginning of her career but increased her motivation sharply in the 2nd and 3rd years. However, her motivation decreased gradually from the 3rd to 7th years but were followed by a rapid increase during the 6th to 8th years.

The last section was a scale with 20 factors that might underlie college EFL teachers’ DTCR. This scale was designed based on questionnaires and qualitative data in former related studies (e.g., Yang et al., 2001; Borg and Liu, 2013; Chen and Wang, 2013; Kim et al., 2014; Peng, 2019). The whole questionnaire was displayed in the Appendix. This questionnaire was firstly reviewed and checked by several EFL English teachers and questionnaire designing experts to ensure its appropriateness. The corrected version was then distributed to participants in this study.

The semi-structured interviews were used to induce solutions to overcome DTCR and remotivate college EFL teachers to conduct research. The interview questions were all revolving around how to motivate them and reduce their DTCR. Since all the interviewees were native Chinese, the interviews were conducted in Chinese to improve communication efficiency. Informed consent was given to each interviewee before the interviews, and the interviews lasted from 17 to 46 min.

### 3.2. Participants

This study adopted simple random sampling to select college EFL teachers in three types of universities^1^ in a city of central China. The paper questionnaires were sent to college English teachers in 10 universities. A total of 400 questionnaires were sent and 339 English teachers returned the questionnaires, among which 318 questionnaires were valid. In valid samples, 216 teachers indicated they had experienced of DTCR, among which 163 (75.46%) participants were female and the other 53 (24.54%) were male teachers. With regards to their age, those teachers ranged from 25 to 66 (mean 37.39, SD 9.77). Specifically, 52 (24.07%) English teachers were in their 20s, 106 (49.07%) were in their 30s, and 58 (26.85%) were in their 40s or above. As for their teaching experience, 75 teachers taught English <5 years, 86 taught 6–10 years, and 55 taught more than 10 years. The majority of them (185 teachers, accounting for 85.65%) hold master’s degrees, and 25 (11.57%) were PhD holders, and the other 6 (2.78%) teachers only hold bachelor’s degrees. Although the participants were teaching English courses in universities, they graduated from different majors: 87 (40.28%) teachers majored in English education or teaching, 55 (25.46%) majored in translation, 39 (18.06%) majored in linguistics, and 29 (13.43%) majored in English literature. While the other 6 (2.78%) teachers were from non-English related majors, like international politics, food science and engineering, management, etc.

This study adopted purposive sampling (Merriam and Tisdell, 2015) and interviewed 12 English teachers and 3 officers administering teachers’ academic research projects in universities. Those participants included ordinary EFL teachers who were fairly demotivated and motivated in research, a PhD supervisor in EFL field, and several research project managers. The basic information of those participants was demonstrated in Table 2.

**Table 2:** Information of the interviewees.

Code	Age	Teaching experience (years)	Degree	University type^a^	Position	Academic title
HB1	43	15	Master’s	PRU	EFL teacher	Associate professor
HB2	36	11	Master’s	PRU	EFL teacher	Associate professor
HB3	38	12	Master’s	PRU	Officer	Lecturer
HB4	41	13	PhD	PRU	EFL teacher	Professor
HB5	49	24	Master’s	PRU	Officer	Professor
HB6	39	12	Master’s	PRU	EFL teacher	Lecturer
DU1	36	11	PhD candidate	OPU	EFL teacher	Associate professor
DU2	34	8	PhD candidate	OPU	EFL teacher	Lecturer
LT1	32	6	Master’s	PRU	EFL teacher	Lecturer
LT2	30	5	Master’s	PRU	EFL teacher	Lecturer
LT3	43	17	Master’s	PRU	Officer	Associate professor
CH	32	6	Master’s	OPU	EFL teacher	lecturer
AH	33	6	Master’s	GPU	EFL teacher	Lecturer
HU	50	25	PhD	GPU	EFL teacher	Professor, PhD supervisor
CU	30	4	Master’s	GPU	EFL teacher	Assistant professor

a PRU means private university; OPU means ordinary public university; GPU means good public university (see Footnote 1).

### 3.3. Data analysis

In this study, 318 valid questionnaires were numbered firstly, and they were the foundation to draw the general motivation change track of EFL teachers. To this end, the motivation level of each teacher in every year was noted and the average motivation level in each year was calculated, and then those average values were dotted and linked on the chart.

In the valid questionnaires, 216 English teachers indicated that they suffered from DTCR in their career. The demotivational reasons in the 216 questionnaires were analyzed by exploratory factor analysis (EFA) to figure out the factors underlying EFL teachers’ DTCR.

In addition, this study interviewed 15 English teachers and officers in charge of teachers’ academic research, and all the interviews were permitted be recorded. The recorded interviews were transcribed into texts by https://www.iflyrec.com/zhuanwenzi.html, and were proofread by the researcher. Those texts were then input into NVivo 12 Plus to analyze. Thematic analysis (Braun and Clarke, 2006) were applied to analyze the 15 files, and they were iteratively read coded, compared, categorized, and integrated.

## 4. Results

### 4.1. Motivation changes for conducting research

**Figure. 2:**
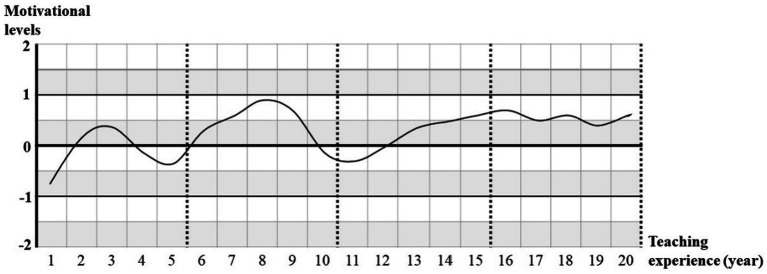
Average curve of EFL teachers’ motivation change. Motivation level 2 = very interesting, 1 = interesting, 0 = neither interesting nor boring, −1 = not interesting, −2 = very boring.

This study drew a general motivation fluctuation graph to demonstrate EFL teachers’ motivation changes of conducting academic research. Every teacher’s motivation value was noted down based on their teaching experience (years), and finally an average curve of those 318 EFL teachers’ motivation levels were drafted (see Figure 2).

This curve demonstrated the general tendency of EFL teachers’ motivation to conduct academic research. It could be found from the curve that teachers’ overall motivation levels were not very high, and the average curve never surpassed 1 during teachers’ 20 years’ teaching experience (Clearly, there were 2 summits of motivation in the curve at the years of 3 and 8. But neither of them exceeded “interesting” level.). In addition, this curve even exhibited that EFL teachers generally had low motivation to conduct academic research in three periods (1st year, 4th to 5th years, and 10th to 12th years) in their career, with motivation level below 0. Apart from the overall motivation levels in each year, this curve also displayed some fluctuations in EFL teachers’ teaching career, especially before the year of 12. For example, there were 2 significant declines in the curve (3rd to 5th year, and 8th to 11th year). Since DTCR covers both low investment and interests in academic research and gradually decreased investment and interests in it, generally 9 years (i.e., 1st year, 3rd to 5th years, and 8th to 12th years) could be named as DTCR years in EFL teachers’ career. Therefore, it could be concluded from the curve that college EFL teachers in China were very likely to suffer from DTCR in their teaching career.

### 4.2. Factors underlying DTCR

Table 3 demonstrated the results of KMO and Bartlett’s test of sphericity. The KMO value reached 0.908, and the Sig. ratio of Bartlett’s test was 0.000 < 0.05. indicating that it was suitable to run factor analysis.

**Table 3:** KMO and Bartlett’s test.

KMO		0.908
Bartlett’s test	Chi-Square	3406.093
	df	190
	Sig.	0.000

This study applied the method of principal components to extract factors based on Eigenvalues greater than 1. In addition, varimax was chosen as the method in rotation. This study extracted 5 components, explaining 78.02% of the total variance. Therefore, the 5 components could reasonably represent the original data. The rotated component matrix was displayed in Table 4. The factors were named based on the contents of the items affiliated to them. For example, item 5, 9 and 20 indicated that EFL teachers’ DTCR was because of their insufficient time, academic resources and social connections. Therefore, the corresponding factors were named as “insufficient research resources.”

**Table 4:** Rotated component matrix.

Factors	Items	Factor loading				
		1	2	3	4	5
1. Weak research ability	Q3	0.811				
	Q2	0.797				
	Q4	0.781				
	Q18	0.771				
	Q1	0.732				
	Q19	0.729				
2. Negative emotions & attitudes	Q14		0.872			
	Q11		0.840			
	Q16		0.829			
	Q17		0.724			
3. Poor research surroundings	Q6			0.816		
	Q8			0.794		
	Q13			0.721		
	Q15			0.683		
4. Research management problems	Q12				0.881	
	Q7				0.870	
	Q10				0.849	
5. Insufficient research resources	Q5					0.841
	Q9					0.804
	Q20					0.793

Extraction method: Principal component analysis, Rotation method: Varimax with Kaiser normalization, Rotation converged in 6 iterations.

Cronbach Alpha coefficient for the reliability of the scale was 0.934. In addition, Cronbach Alpha and Omega coefficients for the five factors were displayed in Table 5.

**Table 5:** Cronbach alpha and omega coefficients for the five factors.

Items	Weak research ability	Negative emotions and attitudes	Poor research surroundings	Research management problems	Insufficient research resources
Cronbach’s *α*	0.939	0.917	0.863	0.918	0.801
Omega	0.940	0.916	0.865	0.921	0.802

These scores showed that Cronbach Alpha and Omega coefficients for each factor were quite high. These results could be regarded as an indicator that the scale was reliable.

The EFA results indicated that EFL teachers were demotivated by five factors for their DTCR, including weak research ability, negative emotions and attitudes, poor research surroundings, research management problems and insufficient resources. These findings demonstrated that, besides EFL teachers’ own factors (i.e., weak research abilities, negative emotions, and attitudes), external factors could also cause their DTCR.

### 4.3. Solutions to overcome DTCR

This study interviewed 12 EFL teachers and 3 officers administrating college teachers’ research and academic projects. The 15 interviews were transcribed into Chinese texts and were analyzed through thematic analysis method (Braun and Clarke, 2006). Based on the steps of thematic analysis, the interview data was firstly coded with blank mind. Overall, 36 initial codes were generated, and those codes were further abstracted into 7 themes. Some examples of coding results and processes were displayed in Table 6.

**Table 6:** Thematic analysis results (excerpt).

Original texts	Codes	Themes	Notes
I have more than 12 classes every week. How can I spare time on research? … I hope our university could reduce my class work.	Reduce class work	teaching load reduction	This means that universities should reduce EFL teachers’ teaching loads. They can allocate teachers less classes or divide positions into teaching and research ones, so that teachers in research positions could have more time on research.
My suggestion is that universities could separate research position from teaching position. In this way, teachers in research position only have very limited classes, and hence could spend more time on research.	Separation of teaching and research position		
I think universities should conduct more research training programs.	Provide training programs for research	appropriate research training provision	Universities should provide some programs to train teachers’ research skills. But universities should notice that the contents should be more specific and well directed. The contents had better focus on foreign languages rather than other fields.
Our universities indeed have conducted several research training programs. But I think the values were limited. For example, a lecture in one program I attended only taught us how to fill in the application form for research funds! However, I want to learn some skills to conduct research. That was a waste of time.	Training program should focus on research skills		
Research training program should pay more attention to the discipline of foreign languages. I have attended many training classes, but many of them were very general. Some of them were even conducted for science and engineering disciplines.	Training program should focus on foreign languages		
I think English teachers only with master’s degree do not have enough ability to conduct academic research. I suggest universities should encourage their teachers to further their studies to get PhD.	Encourage further studies	degree promotion encouragement	PhD programs could give well-round research trainings. Therefore, some officers and teachers encourage EFL teachers’ PhD studies.
I heard *** university provide about 20,000 RMB award for those who publish papers on SSCI indexed journals. I think this could improve teachers’ motivation.	Paper publication incentive	Award and punishment policies	Universities could award those with research motivation while fine those who do not conduct research.
As for some hard science disciplines, many universities require teachers to apply for academic research funds and to publish papers every year. I think this measure could also be used in EFL disciplines. … Specifically, EFL teachers who do not conduct research and publish papers should be fined.	Punishment on those without research production		
I think we should create a research team and focus on one research topic. In this way, we can communicate and discuss with other members and maintain our research motivation.	Research team needed	Research communities constructing	A research community should be constructed. In this community, EFL teachers could communicate their academic ideas and keep motivation.
When I firstly began to conduct research, I just liked a headless chicken. I did not have a fixed research interest then. I think a research leader could help to answer my research problem.	Research leader needed		
It is almost impossible for English teachers in private universities to publish papers in CSSCI indexed journals. Those journals paid too much attention to authors’ degree, academic titles, affiliations. I really hope Chinese journal could truly promote double blind review.	Paper review method reform	Academic fairness promotion	Some unfair practice impedes EFL teachers’ research motivation. Those inappropriate practice should be curbed.
I hope National Social Science Fund could favor teachers in ordinary universities. This is because I found many funds were issues to applicants in good universities.	Fund support to ordinary teachers		
To promote professional titles, some English teachers even buy publication qualification or papers from some editors or agencies. … I think this phenomenon should be prohibited by government.	Academic misconduct prohibition	Illegal research practice prevention	Some teachers resort to abnormal ways to publish papers or get degrees. Those ways are usually illegal.
I think you must have heard about the news recently: some teachers “buy” PhDs from the Philippines. I think this trend should be immediately curbed because those “water PhDs” could not really enhance teachers’ research abilities and motivation.	Illegal foreign PhD programs curb		

**Figure. 3:**
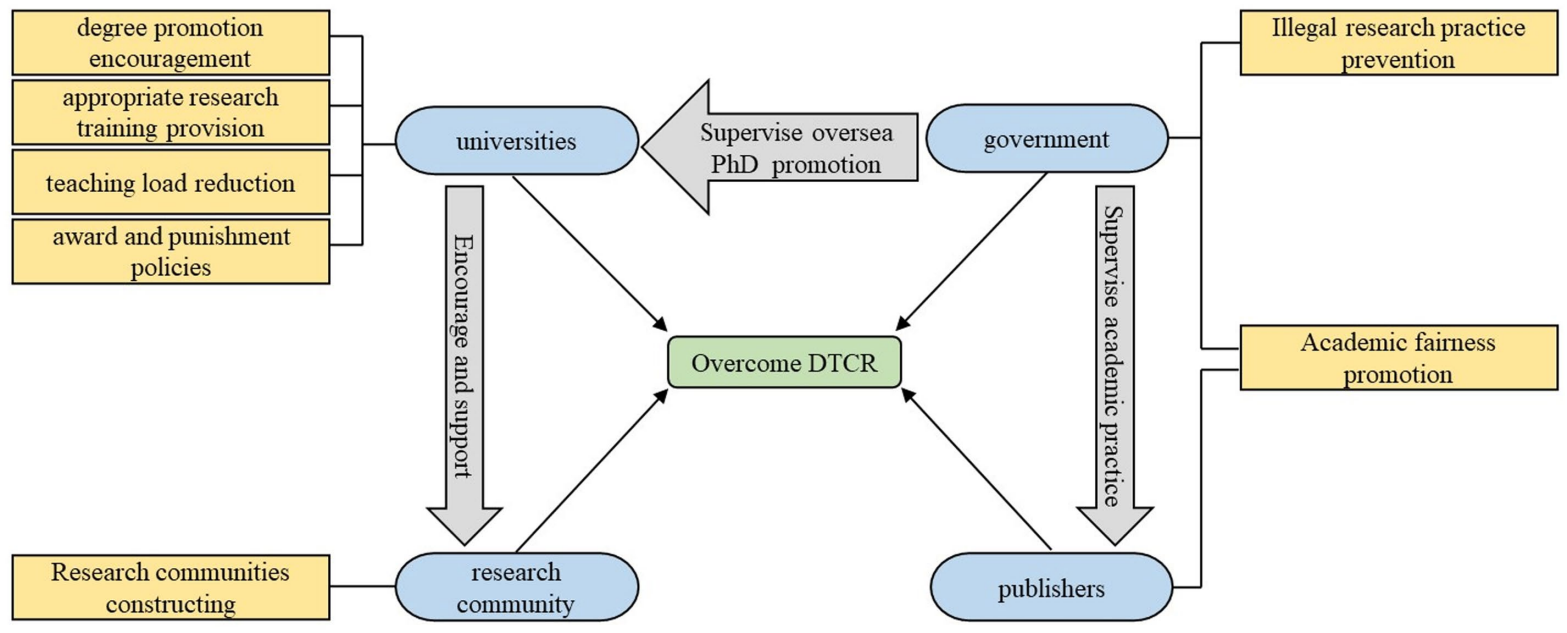
Mind map for thematic analysis results.

From the themes generated from suggestions, it could be concluded that four stakeholders were involved to overcome EFL teachers’ DTCR, including universities, research communities, government, and publishers. After analyzing the underlying logic of the suggestions proposed for overcoming EFL teachers’ DTCR, this study drew a mind map (see Figure 3) to demonstrate the relations between different suggestions.

**Figure. 4:**
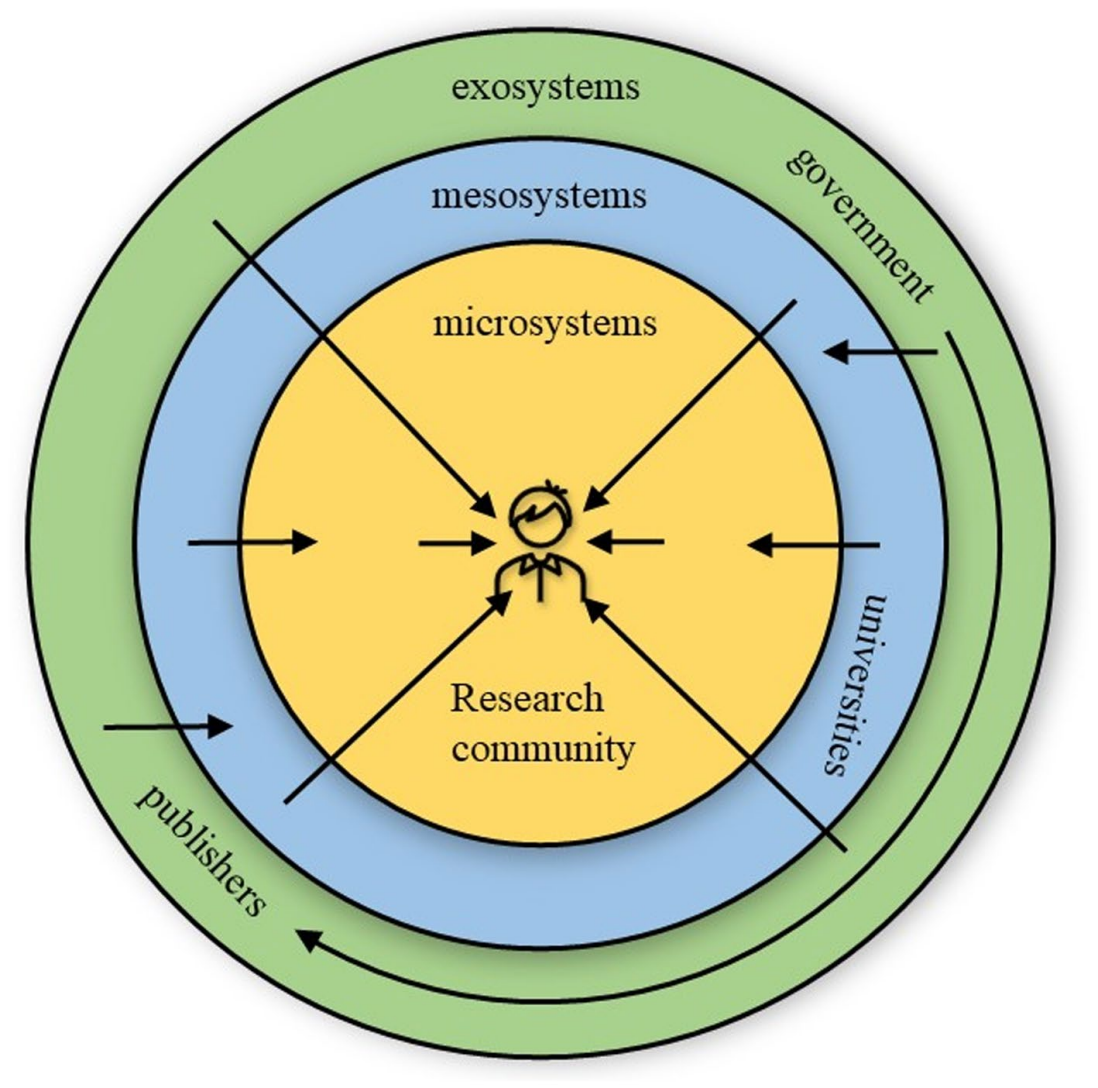
Ecological model for overcoming EFL teachers’ DTCR.

Drawing upon ecological systems theory (Bronfenbrenner, 1979), this study constructed an ecological model to demonstrate the relations among stakeholders who could make efforts for overcoming EFL teachers’ DTCR (Figure 4) based on the thematic analysis results.

This model demonstrated that different stakeholders (including government, publishers, universities, and research communities) in EFL teachers’ working ecology could help them to overcome their DTCR. For example, universities could decrease teachers’ teaching loads to give them more time to conduct research; academic leaders could give advice to novice researchers on their academic activities. What was noteworthy was that measures could also be taken among those stakeholders (except EFL teachers *per se*) to promote teachers’ research behaviors. For instance, government could issue some regulations to prohibit some journals’ academic misconduct, such as selling publication qualification to teachers.

## 5. Discussion

This study investigated the dynamic processes of college EFL teachers’ (de)motivation, explored the factors underlying their DTCR and proposed the solutions to overcome it. It is found that EFL teachers’ motivation to conduct research fluctuated largely and they were very likely to suffer from DTCR in their career. Five factors were found underlying EFL teachers’ DTCR, including weak research ability, negative emotions and attitudes, poor research surroundings, research management problems, and insufficient resources. To help teachers overcome their DTCR, comprehensive measures should be taken by stakeholders in the ecology around EFL teachers, including universities, research communities, government, and publishers.

This study explored college EFL teachers’ motivation changes in their career and found teachers’ motivation levels were generally not high but with large fluctuations. Teachers’ motivation fluctuations in their career echoed with the regulations conferring of professional titles in China. Working period requirements for college teachers with different degrees (i.e., master’s and doctoral) to promote professional titles were almost the same. For example, master’s degree holders should work in universities or colleges for at least 2 years before they could be conferred the title of lecturer and then should work no <5 years to apply the title of associate professor. While PhD holders should work at least 2 years before they could be conferred the title of associate professor and then at least 5 years to be conferred professor title. Because EFL teachers need conduct academic research and publish papers to meet the requirements in the regulations in the specified time period, they tended to motivate themselves. This explained the first summit of the motivation change curve in the second and third years. The underlying reasons were the same for the second summit in the curve, when teachers were preparing for their second academic titles. For EFL teachers, achieving the academic title of professor is difficult and needs more time spent on research (Gao and Li, 2018), therefore their motivation remains comparatively high for long time after the 13th year. According to the motivation dynamic curve, EFL teachers demonstrated significant demotivation in their career in three periods, including 1st year, 3rd to 5th years, and 8th to 12th years. Most of the demotivated periods were just after they got new professional titles. This fluctuation curve of EFL teachers’ DTCR might underlie the “roller coaster” phenomenon in research output of college teachers before and after the title appraisal (Yang and Wang, 2015). Besides, the curve demonstrated that many EFL teachers’ experienced DTCR when they did not need to prepare for the professional titles in the near future, which supported Yuan et al. (2010)’s concern about current annual and tenure checking systems. They thought those systems might not motivate teachers when they are not eager to achieve higher academic titles.

The fluctuated curve of EFL teachers’ research motivation echoed many studies on EFL teachers’ motivation changes. For example, it shared similar findings with Kimura (2014)’s longitudinal case study to investigate EFL teachers’ teaching motivation in Beijing that teachers’ motivation of teaching was also fluctuated. Similar conclusions were also drawn in Song and Kim (2016)’s case studies on two South Korean experienced EFL teachers that EFL teachers’ teaching motivation changed over time. But those study only included quite limited participants in their case study, while the present study demonstrated fluctuations of research motivation were popular among EFL teachers. Though Kim and Kim (2022) investigated (de)motivation dynamics among 144 Korean English teachers and found English teachers with 4–6 years of teaching experience suffered more demotivation in their teaching than teachers in other stages, this study only paid attention to EFL teachers’ motivation fluctuation of conducting research rather than teaching as the other studies did. But overall, those studies (no matter about EFL teachers’ teaching or researching) indicated that a dynamic view should be considered when motivation was investigated.

This study found five factors underlying EFL teachers’ DTCR, including weak research ability, negative emotions and attitudes, poor research surroundings, research management problems and insufficient resources. Those factors involved both internal and external factors. This finding echoed the opinions of Clare et al. (2019) and Sakai and Kikuchi (2009) on demotivation that both internal and external factors should be considered when exploring people’s demotivation. Among the five factors causing EFL teachers’ DTCR, an internal factor, i.e., weak ability, was frequently reported as one reason for demotivation among various groups of people. For example, weak ability (sometimes this factor was named as weak competence, lack of confidence, etc.) was often found to be a factor for students’ demotivation to learn English (e.g., Ghadirzadeh et al., 2012; Ren and Abhakorn, 2022) and teachers’ demotivation to teach English (e.g., Chireshe and Shumba, 2011; Nagamine, 2018). These similar findings supported Bandura (1977)’s self-efficacy theory that people’s subjective evaluation of their abilities could influence their motivation.

This study interviewed several teachers and officers for their suggestions to overcome DTCR, and constructed a model based on their various suggestions. This ecological model echoed Bronfenbrenner (1979)’s ecological systems theory that a person is influenced by different setting s/he lives in, the relations among the settings and even the larger contexts where those settings are included. Although Bronfenbrenner (1979)’s theory emphasizes the influences of ecological systems on children’s development, this study demonstrated that this theory could also be shifted and applied to explain the relations among the stakeholders as to overcome EFL teachers’ DTCR. However, there existed some differences between the model in this study and Bronfenbrenner (1979)’s ecological systems model. First, there are four systems in ecological systems model, including microsystems, mesosystems, exosystems, and macrosystem, while there were only three systems in the model of this study, without the most outside one (i.e., macrosystem). This is because the macrosystem in ecological systems model refers to dominant beliefs and ideologies. Considering the relative stability and consistency of it (Zwicker et al., 2020), the interviewees in this study might thought those invisible values and beliefs could not be changed shortly and largely and do much help to overcome DTCR. Second, ecological systems model exhibited that different settings and contexts could interact with each other, while the relations between different stakeholder in this study were unidirectional. This might be because the interviewees in this study were EFL teachers and officers, without university managers, journal editors or government officials. Those EFL teachers and officers could only unidirectionally sense the influences from the stakeholders in other systems, but their influences on other stakeholders could not be elicited.

## 6. Conclusions and prospects

This study found there existed fluctuations in motivation levels for conducting research among EFL teachers in China, and many of them were very likely to suffer from DTCR in their career. There were five factors causing EFL teachers’ DTCR, including weak research ability, negative emotions and attitudes, poor research surroundings, research management problems, and insufficient resources. To help teachers overcome their DTCR, comprehensive measures should be taken by stakeholders in the ecology around EFL teachers. Specifically, except for college EFL teachers themselves, this study also provided implications for other entities, including universities, research communities, government, and publishers that they could make their corresponding efforts to help EFL teachers to overcome their DTCR. For example, universities could decrease teachers’ teaching loads to give them more time to conduct research; government could issue some regulations to prohibit some journals’ academic misconduct, such as selling publication qualification to teachers.

A motivation change graph was demonstrated in this study to showcase the dynamicity of college EFL teachers’ motivation to conduct research. Although the method of drawing graphs was often applied in students’ motivational research, this method was seldom used in studies among other groups of people, e.g., EFL teachers. This study improved this method and exhibited its good validity. Therefore, future studies could utilize this method to explore motivational dynamicity among different groups of people.

This study found EFL teachers tended to suffer from DTCR in some periods of years (i.e., 1st year, 3rd to 5th years, and 8th to 12th years), and investigated the reasons underlying DTCR. However, the factors for DTCR in different periods might be differentiated. For example, the factors for EFL teachers’ DTCR in the first year were very likely different from those for 8th to 12th years. Therefore, future studies could be conducted to investigate and compare the factors for DTCR in particular periods and explore the reasons underlying the differences.

This study constructed a model to demonstrate the stakeholders who should make their efforts to help overcome college English teachers’ DTCR by interviewing college English teachers and officers managing college teachers’ research. Although the model involved universities, government, and publishers in different systems, this study did not interview people in those sectors and triangulate the model. Therefore, these people should be included, and the influences of the interactions between different stakeholders on EFL teachers’ motivation to conduct research should be investigated in future DTCR related research.

## 7. Limitations

To answer the questions in the questionnaire applied in this study, participants needed to retrospect their past research experience. Considering some teachers had long working experience (e.g., some had more than 10 years), they might not remember very clearly of their research motivation features in a specific year, especially in such a limited time for filling the questionnaire.

## Data availability statement

The raw data supporting the conclusions of this article will be made available by the authors, without undue reservation.

## Ethics statement

Ethical review and approval was not required for the study on human participants in accordance with the local legislation and institutional requirements. The patients/participants provided their written informed consent to participate in this study.

## Author contributions

XR was responsible for designing the research, analyzing the data, and writing the original manuscript. FZ collected the data, and provided many suggestions for revising the manuscript.

## Funding

This research was funded by Hubei Business College with the academic project name of “ A Study of Demotivation to Learn English among College Students in China’s Private Universities” (item code: KY202141). In addition, this study was supported by the fund of provincial first-class majors issued by Ministry of Education of China. Specifically, the provincial first-class major supporting this study was “English” undertaken by Hubei Business College.

## Conflict of interest

The author declares that the research was conducted in the absence of any commercial or financial relationships that could be construed as a potential conflict of interest.

## Publisher’s note

All claims expressed in this article are solely those of the authors and do not necessarily represent those of their affiliated organizations, or those of the publisher, the editors and the reviewers. Any product that may be evaluated in this article, or claim that may be made by its manufacturer, is not guaranteed or endorsed by the publisher.

## Footnotes

^1^In China, universities are operated by different entities, including government and companies. Thus, they could be divided into public universities and private universities. Usually, public universities are ranked higher than private ones, and they are operated with different standards of quality. Generally, the universities listed in the "double first-class" university project (Ministry-of-Education, 2022) boast better student sources and education quality than those excluded from the project. Based on those considerations, the three different types of universities were identified as: private university (PRU), ordinary public university (OPU), and good public university (GPU) in this study.
