# Supplementary material for: College EFL teachers’ demotivation to conduct research: A dynamic and ecological view
Source: Front Psychol. 2023 Jan 19;13:1071502. doi: 10.3389/fpsyg.2022.1071502 (PMC9893777; doi:10.3389/fpsyg.2022.1071502)
Supplement: Supplementary file 1 [file Data_Sheet_1.docx]

**Appendix**

**(Translated version)**

**Questionnaire for demotivation to conduct**

**academic research among college EFL students***

**Section 1**

age:

gender: □female □male

teaching experience: years

major:

degree:

course teaching:

**Section 2**

Have you experienced DTCR in your teaching career?

□ Yes

□ No

Please draw a line or curve to indicate your motivation changes during your teaching career.


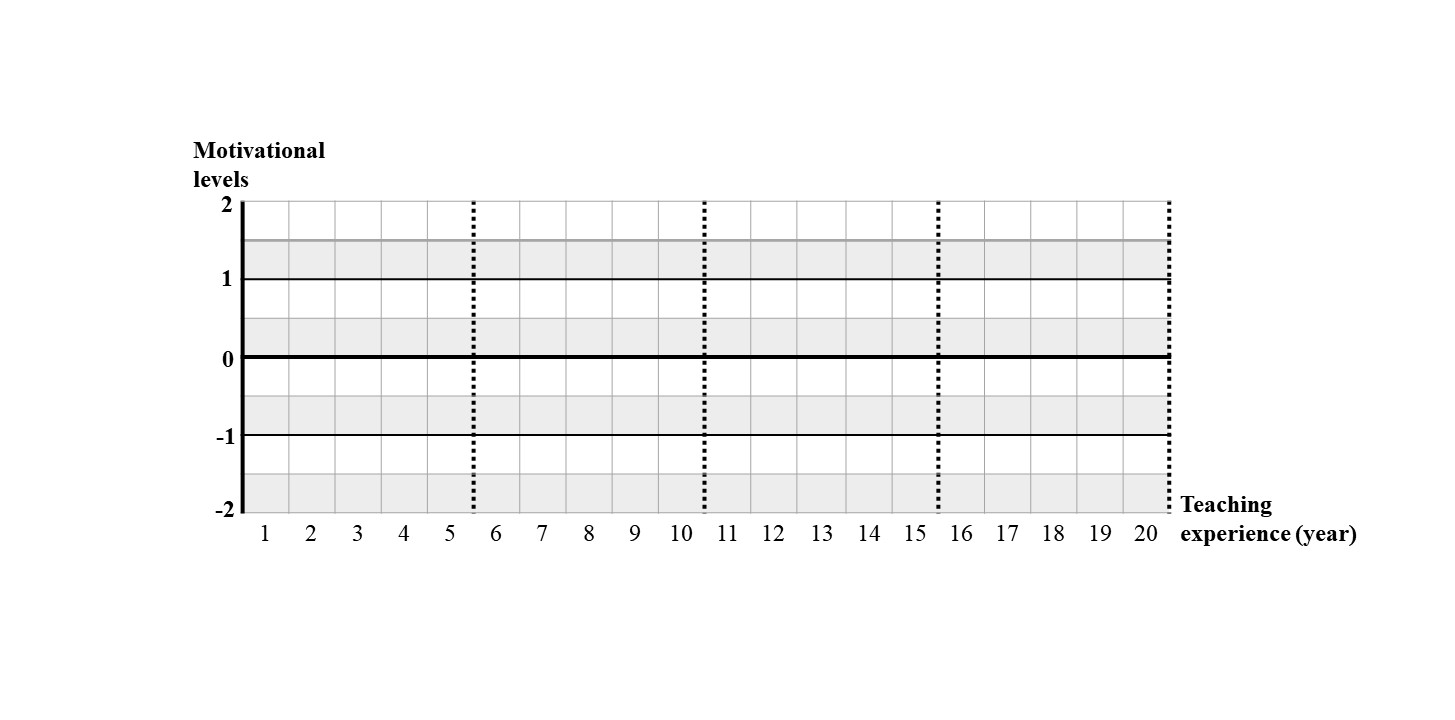


Note: motivation level 2 = very interesting, 1 = interesting, 0 = neither interesting nor boring, -1 = not interesting, -2 = very boring.

**Section 3**

The following scale displays some factors underlying college EFL teachers’ demotivation to conduct academic research. Please fill in the questionnaire based on your real situation.

1. I lack understanding of academic publication.
2. I lack understanding of the frontiers and trends of my research fields.
3. I lack experience of writing academic papers.
4. I did not grasp research methods systematically.
5. Taking care of my family occupies too much time for research.
6. My department lacks academic leaders and research teams.
7. Our university emphasizes teaching rather than research.
8. My colleagues seldom conduct research.
9. The university I am working in does not provide enough academic resources.
10. School managers apply management methods of science and engineering to foreign language fields.
11. I lack interests in research.
12. Our department lack managers to organize research.
13. My university gives me too much administrative work.
14. I think the value of conducting research in foreign language fields is limited.
15. My university arranges me too many classes to teach.
16. Research papers are too obscure.
17. I gain little despite much research input.
18. My education background and affiliation are not good enough to apply research funds.
19. My education background and affiliation are not good enough to publish high-quality papers.
20. The high-quality paper publication needs many social connections.

* There is an open letter at the beginning of the questionnaire, introducing the researcher’s information, aims of the questionnaire and the inform consent. For the 20 items, there are 7-point scales following each of them in the following way:

□strongly agree □agree □slightly agree □not sure □slightly disagree □disagree □strongly disagree

At the end of the questionnaire, there is a closing sentence to thank the participants.
